# The HMGB1 (C106A) mutation inhibits IL-10-producing CD19^hi^FcγRIIb^hi^ B cell expansion by suppressing STAT3 activation in mice

**DOI:** 10.3389/fimmu.2022.975551

**Published:** 2022-08-02

**Authors:** Mengru Liu, Jingwen Zhou, Rui Yin, Hui Yin, Yue Ding, Feng Ma, Li Qian

**Affiliations:** ^1^ Institute of Translational Medicine, Medical College, Yangzhou University, Yangzhou, China; ^2^ CAMS Key Laboratory of Synthetic Biology Regulatory Elements, Institute of Systems Medicine, Chinese Academy of Medical Sciences and Peking Union Medical College, Beijing, China; ^3^ Suzhou Institute of Systems Medicine, Suzhou, China; ^4^ Jiangsu Key Laboratory of Integrated Traditional Chinese and Western Medicine for Prevention and Treatment of Senile Diseases, Yangzhou, China

**Keywords:** HMGB1, CD19^hi^FcγRIIb^hi^ B cells, STAT3, sepsis, IL-10

## Abstract

Regulatory B cells have important roles in inflammation and autoimmune diseases. A newly discovered subpopulation of B cells with a CD19^hi^FcγRIIb^hi^ phenotype inhibits the proliferation of CD4^+^ T cells by secreting interleukin (IL)-10. The expansion of CD19^hi^FcγRIIb^hi^ B cells in mouse spleen can be induced by lipopolysaccharide (LPS) or CpG oligodeoxynucleotide stimulation. However, the mechanism of CD19^hi^FcγRIIb^hi^ B cell expansion and its role in inflammatory diseases are unclear. Here, we report that, under inflammatory conditions, the proliferation and immunosuppressive function of CD19^hi^FcγRIIb^hi^ B cells were decreased in high mobility group box1 (HMGB1) C106A mutant mice, compared with wild-type mice. The HMGB1 (C106A) mutation in B cells reduced STAT3 phosphorylation, restricting the expansion and suppressive function of CD19^hi^FcγRIIb^hi^ B cells. Compared with CD19^hi^FcγRIIb^hi^ B cells from wild-type mice, CD19^hi^FcγRIIb^hi^ B cells from *Hmgb1* ^(C106A)^ mice significantly reduced the survival of mice with sepsis. Recombinant HMGB1 promoted the expansion of IL-10-producing CD19^hi^FcγRIIb^hi^ B cells among LPS-activated B cells *in vitro*. Furthermore, the percentage of CD19^hi^FcγRIIb^hi^ regulatory B cells in the peripheral blood was increased in patients with sepsis, compared with healthy controls. These findings implicate the role of HMGB1 in the expansion and immunosuppressive function of CD19^hi^FcγRIIb^hi^ B cells.

## Introduction

Regulatory B cells (Bregs) constitute a unique type of B cells with immunomodulatory functions that maintain immune tolerance and suppress harmful immune responses (1). Bregs suppress immune responses by inhibiting the activation and differentiation of dendritic cells; CD4^+^, CD8^+^, and NK T cells; and effector T (Th1 or Th17) cells. Their suppression mechanism also includes inducing the generation of regulatory T cells (CD4^+^CD25^+^Foxp3^+^ Tregs) (2). Bregs maintain tolerance and inhibit inflammation and autoimmunity by producing interleukin (IL)-10, transforming growth factor-β (TGF-β), or IL-35; thus, they have important roles in inflammation-related diseases (3).

IL-10-producing Bregs have an immunosuppressive role in the pathogenesis of several diseases (4). In mice, IL-10-producing Bregs have several phenotypes, including CD19^+^CD5^+^CD1d^hi^ (5) and CD19^+^Tim-1^+^ (6). In humans, several subsets of Bregs inhibit effector T cells by producing IL-10; these subsets include transitional CD19^+^CD24^hi^CD38^hi^ B cells, CD19^+^CD24^hi^CD27^+^ B cells (7), and Tim-1^+^ B cells (8). Whereas regulatory T cells are known to express the transcription factor foxp3, there are no known transcription factors responsible for Bregs differentiation or development (9). A subset of Bregs, CD19^hi^FcγRIIb^hi^ B cells, inhibits CD4^+^ T cell proliferation by secreting IL-10 (10). CD19^hi^FcγRIIb^hi^ B cells are expanded in mouse spleen after stimulation with lipopolysaccharide (LPS) or CpG oligodeoxynucleotides (ODN). However, the mechanism of CD19^hi^FcγRIIb^hi^ B cell expansion and its role in inflammatory diseases are unclear.

High mobility group box1 (HMGB1) is a nuclear protein named for its rapid migration during electrophoresis (11). It is expressed in all eukaryotic cells and is highly evolutionarily conserved. HMGB1 has three conserved cysteine residues at positions 23, 45, and 106 (12). HMGB1 is a damage-associated molecular pattern protein involved in several disease states, including cancer (13), arthritis (14), and sepsis (15); it also acts as a late mediator in endotoxemia. HMGB1 is actively secreted by monocytes and macrophages after stimulation with microbes or proinflammatory cytokines (11). HMGB1 affects multiple immune cells; it inhibits the proliferation and differentiation of T cells (16), enhances the function of regulatory T cells (17), induces the production of myeloid-derived suppressor cells (18), and activates NK cells (19). Here, we hypothesized that HMGB1 has a regulatory role in CD19^hi^FcγRIIb^hi^ B cells.

We constructed *Hmgb1* ^(C106A)^ mice in a C57BL/6 background, such that the cysteine at nucleotide 106 in Hmgb1 was replaced by alanine; this led to suppression of Hmgb1 function (20, 21). We used *Hmgb1* ^(C106A)^ mice to investigate the role of HMGB1 in CD19^hi^FcγRIIb^hi^ B cells during an inflammatory response and explore the underlying molecular mechanisms.

## Materials and methods

### Mice

C57BL/6 mice (female, 6–8 weeks) were obtained from the Experimental Animal Center of Yangzhou University (Yangzhou, China). *Hmgb1* mutant mice (*Hmgb1* ^(C106A)^) on a C57BL/6 background were obtained from Biocytogen (Beijing, China). A pair of sgRNAs (5’-GTGATCTCAAGagtagGcacAGG-3’ and 5’-GGTAACTGTCattagaATGGAGG-3’) was designed to produce a 6.4-kb deletion in the EGE-ZJH-024 gene (GENE ID: 15289). Next, cas9/sgRNA mRNA was co-microinjected into the zygote using a targeted vector with one C106A mutation site, two homologous sequences, and two *Lox*P sites. The sequences of the mouse genotyping primers were 5’-TTAGGTGCACGAGGGGCACTTATTG-3’ and 5’-AGCTAGGCAGAACACTGCAAACCAT-3’. The polymerase chain reaction products from wild-type (WT) and *Hmgb1* ^(C106A)^ mutant mice were 394 bp and 479 bp, respectively. The experimental animal procedures were approved by the Animal Care and Use Committee of Yangzhou University (Yangzhou, China).

### Patient samples

The Ethics Committee of the Medical College of Yangzhou University approved this study protocol. Peripheral blood samples from healthy donors and patients with sepsis were obtained from Subei People’s Hospital, which is affiliated with Yangzhou University (**Table S1**). Patients with concurrent human immunodeficiency virus infection, cancer, or autoimmune disease were excluded. Peripheral blood mononuclear cells (PBMCs) were isolated from blood samples by Ficoll centrifugation. CD19^hi^FcγRIIb^hi^ B, CD19^low^FcγRIIb^low^ B, or CD4^+^ T cells from PBMCs were sorted using a FACS Aria SORP (BD Biosciences) with anti-human CD19, FcγRIIb, or CD4 antibodies. Peripheral blood (2 mL) from 40 healthy donors and 44 patients with sepsis was used to isolate PBMCs for flow cytometry. Peripheral blood (20 mL) from three healthy donors and three patients with sepsis was used to isolate CD19^hi^FcγRIIb^hi^ and CD19^low^FcγRIIb^low^ B cells for enzyme-linked immunosorbent assays (ELISAs). Peripheral blood (20 mL) from three healthy donors and three patients with sepsis was used to isolate CD19^hi^FcγRIIb^hi^ and CD19^low^FcγRIIb^low^ B cells for coculture.

### Treatment of mice

To establish a model of inflammation, mice were intraperitoneally injected with LPS (*Escherichia coli* 055:B5; Sigma-Aldrich, 10 mg/kg) or pre-inoculated with D-GalN (600 mg/kg bodyweight) for 1 h, then injected with CpG ODN (10 nmol/mouse) (22). Seven days after injection, mice were euthanized.

### Cell purification

Mouse spleens were collected aseptically, then incubated in red blood cell lysis buffer for 5 min. Next, single-cell suspensions of mouse splenocytes were stained using anti-mouse CD19 beads. CD19^+^B cells were isolated from erythrocyte-depleted splenocytes using anti-mouse CD19 beads (Miltenyi Biotech), in accordance with the manufacturer’s instructions. In some experiments, CD19^hi^FcγRIIb^hi^ B cells and CD19^low^FcγRIIb^low^ B cells were sorted using a FACS Aria SORP (BD Biosciences). B cell purity was typically > 90%. Splenic CD4^+^ T cells were enriched using anti-CD4 beads (Miltenyi Biotech). CD4^+^ T cell purity was typically > 95%.

### Flow cytometry

The fluorochrome-conjugated antibodies used in this study are shown in **Table S2**. Cells were collected, stained with antibodies, and subjected to flow cytometry (FACS Calibur or FACS Verse, BD Biosciences) for surface markers. The results were analyzed using FlowJo 10.0 software. Absolute cell counts were determined using CountBright™ Absolute Counting Beads (Invitrogen Inc). The cell concentration (cells/μL) was calculated using the equation: cells/μL = number of cell events ÷ number of bead events × assigned bead count of the lot/volume of sample (μL). For intracellular staining, cells were first stained with surface markers for 20 min, then fixed and permeabilized using the Foxp3/Transcription Factor Staining Buffer Kit (Cat No. 00-5523-00, eBioscience) for 40 min; finally, they were stained with an anti-Ki67 antibody for 20 min. Samples were resuspended in 400 μL of Permeabilization Buffer (1×) and analyzed by flow cytometry.

### ELISA of cytokines

Mouse IL-10 levels were measured using Mouse IL-10 ELISA kit (Cat No. M1000B, R&D Systems) or Mouse IL-10 ELISA kit (Cat No. 1211002, DRKEWE), in accordance with the manufacturer’s protocol. Human IL-10 levels in plasma were measured using Human IL-10 ELISA kit (Cat No. EK100HS-96, MULTI SCIENCES), in accordance with the manufacturer’s protocol. Human HMGB1 levels in plasma were measured using Human HMGB1 ELISA kit (Cat No. SEKH-0409, Solarbio), in accordance with the manufacturer’s protocol.

### *In vitro* induction of CD19^hi^FcγRIIb^hi^ B cell expansion

CD19^+^ B cells (2 × 10^5^/well) isolated from mouse spleens were treated or not with LPS (Sigma-Aldrich, 10 μg/mL) for 2 days. The cells were harvested and analyzed by flow cytometry. In some experiments, CD19^+^ B cells were pretreated with a specific inhibitor of STAT3 (cucurbitacin I, 500 nM), then exposed to the indicated stimuli. In some experiments, CD19^+^ B cells (2 × 10^5^/well) isolated from mouse spleens were treated or not with recombinant human HMGB1 (R&D Systems, 10 μg/mL), LPS (Sigma-Aldrich, 500 ng/mL), or CpG ODN (0.02 μM) for 2 days.

### CD4^+^T cell proliferation assay

CD4^+^ T cells were seeded into 96-well round-bottom plates precoated with 5 μg/mL anti-CD3 (BioLegend) in the presence of 5 μg/mL soluble anti-CD28 (BioLegend) antibody. CD19^hi^FcγRIIb^hi^ B cells were cocultured with CD4^+^ T cells at a 1:1 ratio. In some experiments, 10 μg/mL neutralizing anti-IL-10 antibody (eBioscience) or 5 μg/mL recombinant IL-10 (rIL-10) protein (PeproTech) was added to the culture medium. After 48 h, cells were stained for CD4 and Ki67, then analyzed by flow cytometry (23).

### Western blotting

Total protein extracts were prepared using a Whole Cell Lysis Assay kit (KeyGen, China), resolved *via* sodium dodecyl sulfate–polyacrylamide gel electrophoresis, and transferred onto polyvinylidene fluoride membranes. The membranes were blocked with bovine serum albumin. After they had been stained with primary and secondary antibodies, the membranes were imaged and analyzed using Compass software.

### Statistics

Data are shown as means ± standard errors of the mean (SEM). For comparisons between two groups, data were analyzed by Student’s *t*-test or the Mann–Whitney test. Survival curves were generated with GraphPad Prism 8 software, and survival analysis was performed using the log-rank (Mantel–Cox) test. Significant differences are expressed as follows: *, P < 0.05; **, P < 0.01; and ***, P < 0.001.

## Results

### Decreased percentage and number of CD19^hi^FcγRIIb^hi^ B cells in *Hmgb1* ^(C106A)^ mice

**Figure 1:**
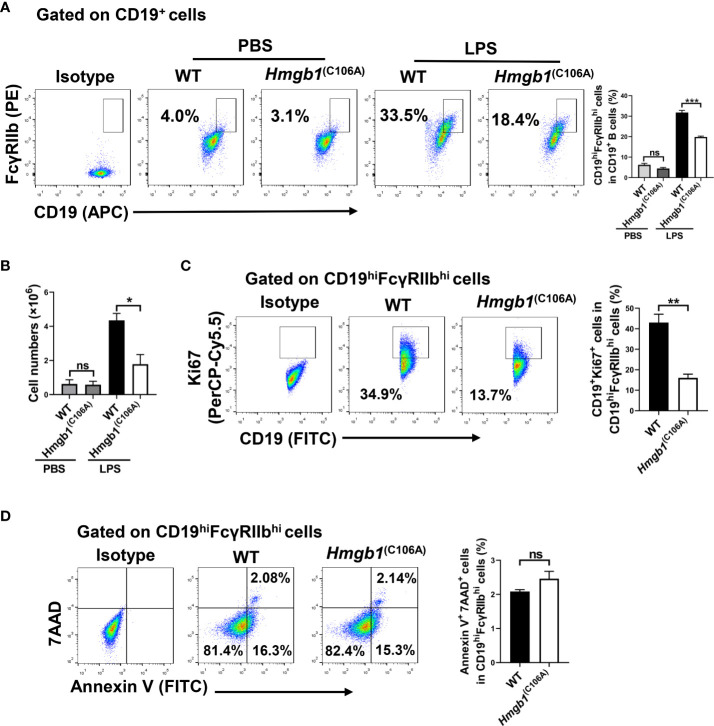
Decreased CD19^hi^FcγRIIb^hi^ B cell percentage and number in *Hmgb1* ^(C106A)^ mice. PBS or LPS was injected into WT and *Hmgb1* ^(C106A)^ mice, and the mice were euthanized 7 days later. **(A)** Percentage and **(B)** number of CD19^hi^FcγRIIb^hi^ B cells in the spleen, analyzed by flow cytometry. Data are shown as means ± SEMs of three independent experiments. **(C)** Percentage of proliferating (Ki67^+^) CD19^hi^FcγRIIb^hi^ B cells in WT and *Hmgb1* ^(C106A)^ mouse spleens, analyzed by flow cytometry with Ki67 staining. Data are shown as means ± SEMs of three independent experiments. **(D)** Representative staining and percentage of AnnexinV^+^7AAD^+^ cells among CD19^hi^FcγRIIb^hi^ B cells from WT and *Hmgb1* ^(C106A)^ mouse spleens, analyzed by flow cytometry. Data are shown as means ± SEMs of three independent experiments. NS, not significant; *P < 0.05; **P < 0.01; ***P < 0.001.

WT and *Hmgb1* ^(C106A)^ mice were injected with phosphate-buffered saline (PBS) or LPS (10 mg/kg), and the percentages of splenic immune cells were determined 7 days later. The percentages of splenic immune cells—including CD3^+^ T cells, CD19^+^ B cells, NK cells, CD4^+^CD25^+^Foxp3^+^ Tregs, and macrophages—did not differ between WT and *Hmgb1* ^(C106A)^ mice, irrespective of LPS-induced inflammation (**Figure S1A**). Although the percentages of CD19^hi^FcγRIIb^hi^ B cells in WT and *Hmgb1* ^(C106A)^ mice did not differ after PBS injection, the percentage of CD19^hi^FcγRIIb^hi^ B cells was lower in the spleen of *Hmgb1* ^(C106A)^ mice with LPS-induced inflammation, compared with the percentage in WT mice (**Figure 1A**). In addition, the percentage of CD19^hi^FcγRIIb^hi^ B cells in lymph nodes did not differ between WT and *Hmgb1* ^(C106A)^ mice with LPS-induced inflammation (**Figure S2**). Next, WT and *Hmgb1* ^(C106A)^ mice were pre-inoculated with D-GalN (600 mg/kg) for 1 h and injected with PBS or CpG ODN (10 nmol/mouse); the percentages of splenic immune cells were determined 7 days later. The percentage of CD19^hi^FcγRIIb^hi^ B cells was decreased in the spleen of *Hmgb1* ^(C106A)^ mice with CpG ODN-induced inflammation, compared with the percentage in WT mice (**Figure S3**). Moreover, flow cytometry showed that the number of CD19^hi^FcγRIIb^hi^ B cells in the spleen of *Hmgb1* ^(C106A)^ mice with LPS-induced inflammation was decreased, compared with the number in WT mice (**Figure 1B**). Because inflammatory *Hmgb1* ^(C106A)^ mice exhibited a decreased number of splenic CD19^hi^FcγRIIb^hi^ B cells, we evaluated the role of Hmgb1 in proliferation or apoptosis of CD19^hi^FcγRIIb^hi^ B cells. WT and *Hmgb1* ^(C106A)^ mice injected with LPS were euthanized 7 days after injection. The percentages of proliferating (Ki67^+^) and apoptotic (AnnexinV^+^7AAD^+^) CD19^hi^FcγRIIb^hi^ B cells in WT and *Hmgb1* ^(C106A)^ mouse spleens were analyzed by flow cytometry. The proliferation of CD19^hi^FcγRIIb^hi^ B cells from LPS-induced inflammatory *Hmgb1* ^(C106A)^ mice was decreased, compared with proliferation in WT mice (**Figure 1C**). However, there was no significant difference in the apoptosis of CD19^hi^FcγRIIb^hi^ B cells between WT and *Hmgb1* ^(C106A)^ mice (**Figure 1D**). Taken together, these results demonstrate that the HMGB1 (C106A) mutation inhibited CD19^hi^FcγRIIb^hi^ B cell expansion in mice with LPS-induced inflammation.

### CD19^hi^FcγRIIb^hi^ B cells from *Hmgb1* ^(C106A)^ mice with inflammation exhibit decreased suppressive activity

**Figure 2:**
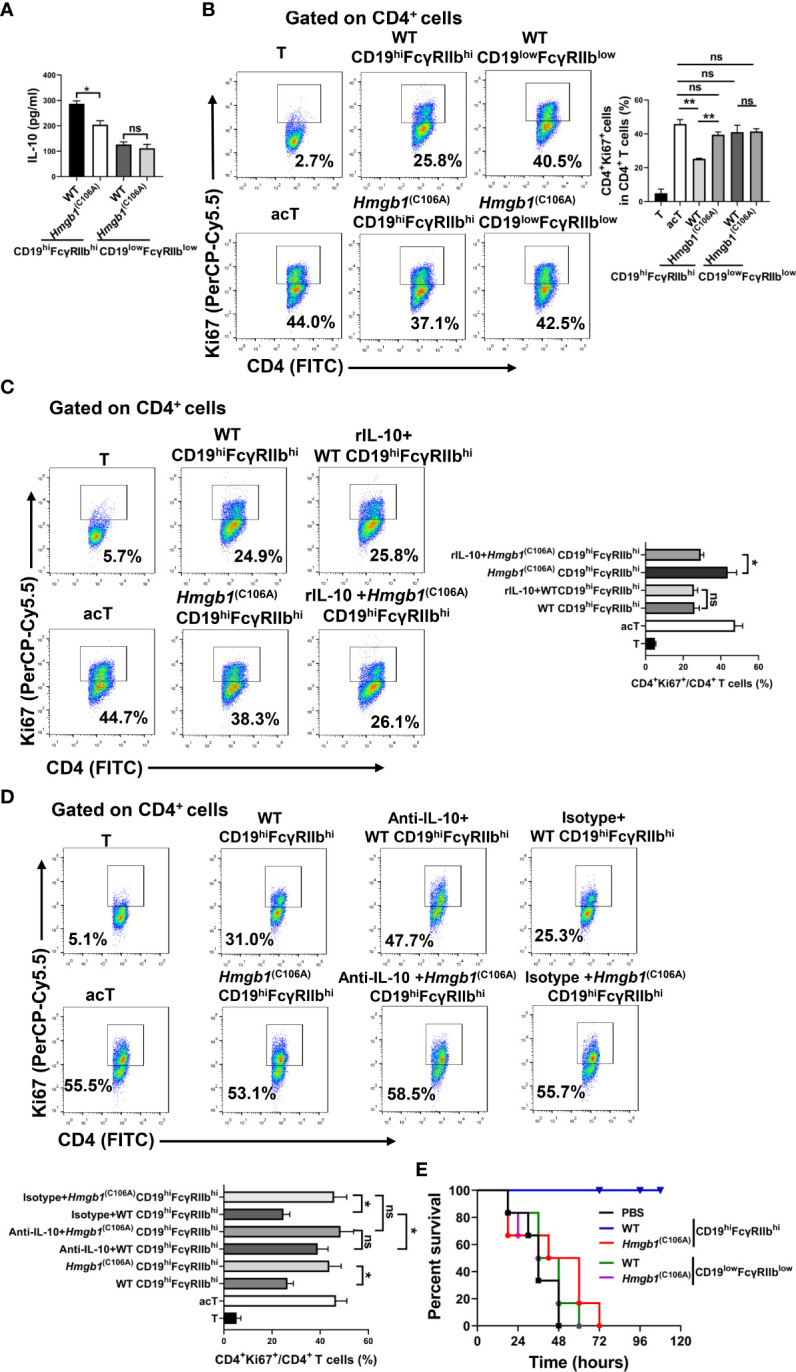
*Hmgb1* ^(C106A)^ inhibited the suppressive function of CD19^hi^FcγRIIb^hi^ B cells in mice. WT and *Hmgb1* ^(C106A)^ mice were injected with LPS on day 0. On day 7, splenic CD19^hi^FcγRIIb^hi^ B cells and CD19^low^FcγRIIb^low^ B cells were sorted by flow cytometry. **(A)** CD19^hi^FcγRIIb^hi^ B cells or CD19^low^FcγRIIb^low^ B cells (2 × 10^5^) were plated on 96-well plates. After 24 h, supernatants were collected for measurement of IL-10 levels by ELISA. Data are shown as means ± SEMs of three independent experiments. **(B–D)** Purified CD19^hi^FcγRIIb^hi^ B cells (2 × 10^5^) from LPS-treated mice were cocultured with anti-CD3/CD28-activated CD4^+^ T cells (2 × 10^5^) **(B)** in the presence or absence of rIL-10 **(C)** or anti-IL-10 **(D)**. After 48 h, CD4^+^ T cell proliferation was determined by Ki67 staining and flow cytometry. Representative histograms show Ki67 expression by CD4^+^ T cells and the proliferation of CD4^+^ T cells. Data are shown as means ± SEMs of three independent experiments. **(E)** C57BL/6 mice were injected with purified CD19^hi^FcγRIIb^hi^ B cells or CD19^low^FcγRIIb^low^ B cells from LPS-treated WT and *Hmgb1* ^(C106A)^ mice; PBS served as the control condition. After 1.5 h, LPS (15 mg/kg) was injected into the mice to induce sepsis, and mortality was monitored. Survival analysis was performed by log-rank (Mantel–Cox) tests; n = 6 mice per group. *P < 0.05; **P < 0.01; NS, not significant.

We investigated whether the HMGB1 (C106A) mutation affected IL-10 and TGF-β production by CD19^hi^FcγRIIb^hi^ B cells. WT and *Hmgb1* ^(C106A)^ mice were injected with LPS; splenic CD19^hi^FcγRIIb^hi^ B cells and CD19^low^FcγRIIb^low^ B cells were sorted by flow cytometry on day 7. CD19^hi^FcγRIIb^hi^ B cells or CD19^low^FcγRIIb^low^ B cells (2 × 10^5^) were plated in 96-well plates for 24 h; then, the IL-10 and TGF-β levels in culture supernatant were assayed. There was no significant difference in the secretion of TGF-β by CD19^hi^FcγRIIb^hi^ B cells between WT and *Hmgb1* ^(C106A)^ mice with LPS-induced inflammation (**Figure S4**). However, *Hmgb1* ^(C106A)^ splenic CD19^hi^FcγRIIb^hi^ B cells from mice with LPS-induced inflammation exhibited lower IL-10 secretion, compared with those cells from WT mice (**Figure 2A**).

WT and *Hmgb1* ^(C106A)^ mice were injected with LPS; splenic CD19^hi^FcγRIIb^hi^ B cells and CD19^low^FcγRIIb^low^ B cells were sorted by flow cytometry on day 7. Purified CD19^hi^FcγRIIb^hi^ B cells or CD19^low^FcγRIIb^low^ B cells (2 × 10^5^) were cocultured with anti-CD3/CD28-activated CD4^+^ T cells (2 × 10^5^). The proliferation of CD4^+^ T cells was evaluated by flow cytometry after 2 days. The ability of CD19^hi^FcγRIIb^hi^ B cells from *Hmgb1* ^(C106A)^ mice with LPS-induced inflammation to inhibit the proliferation of activated CD4^+^ T cells was significantly decreased, compared with that ability in cells from WT mice (**Figure 2B**). To determine whether the effect of the HMGB1 (C106A) mutation on the CD19^hi^FcγRIIb^hi^ B cell-mediated inhibition of CD4^+^ T cells was caused by reduced secretion of IL-10, we added rIL-10 or anti-IL-10 antibody to cocultures of activated CD4^+^ T cells and CD19^hi^FcγRIIb^hi^ B cells. rIL-10 enhanced the *Hmgb1* ^(C106A)^ CD19^hi^FcγRIIb^hi^ B cell-mediated inhibition of T cell proliferation (**Figure 2C**). The anti-IL-10 antibody reduced the ability of WT CD19^hi^FcγRIIb^hi^ B cells to inhibit T cell proliferation (**Figure 2D**). Therefore, the HMGB1 (C106A) mutation reduced the CD19^hi^FcγRIIb^hi^ B cell-mediated inhibition of CD4^+^ T cells by reducing IL-10 secretion in mice with LPS-induced inflammation.

We investigated the role of CD19^hi^FcγRIIb^hi^ B cells from *Hmgb1* ^(C106A)^ mice in LPS-induced inflammation. WT and *Hmgb1* ^(C106A)^ mice were injected with LPS; splenic CD19^hi^FcγRIIb^hi^ B cells and CD19^low^FcγRIIb^low^ B cells were sorted by flow cytometry on day 7. C57BL/6 mice were injected with purified CD19^hi^FcγRIIb^hi^ B cells or CD19^low^FcγRIIb^low^ B cells; PBS served as the control condition. After 1.5 h, LPS (15 mg/kg) was injected into the mice to induce acute sepsis, and mortality was monitored. Compared with WT mice, CD19^hi^FcγRIIb^hi^ B cells from *Hmgb1* ^(C106A)^ mice significantly reduced the survival of mice with sepsis (**Figure 2E**). Therefore, the HMGB1 (C106A) mutation inhibited the suppressive function of CD19^hi^FcγRIIb^hi^ B cells in mice with LPS-induced inflammation.

### Decreased expansion and suppressive function of CD19^hi^FcγRIIb^hi^ B cells among *Hmgb1* ^(C106A)^ B cells

**Figure 3:**
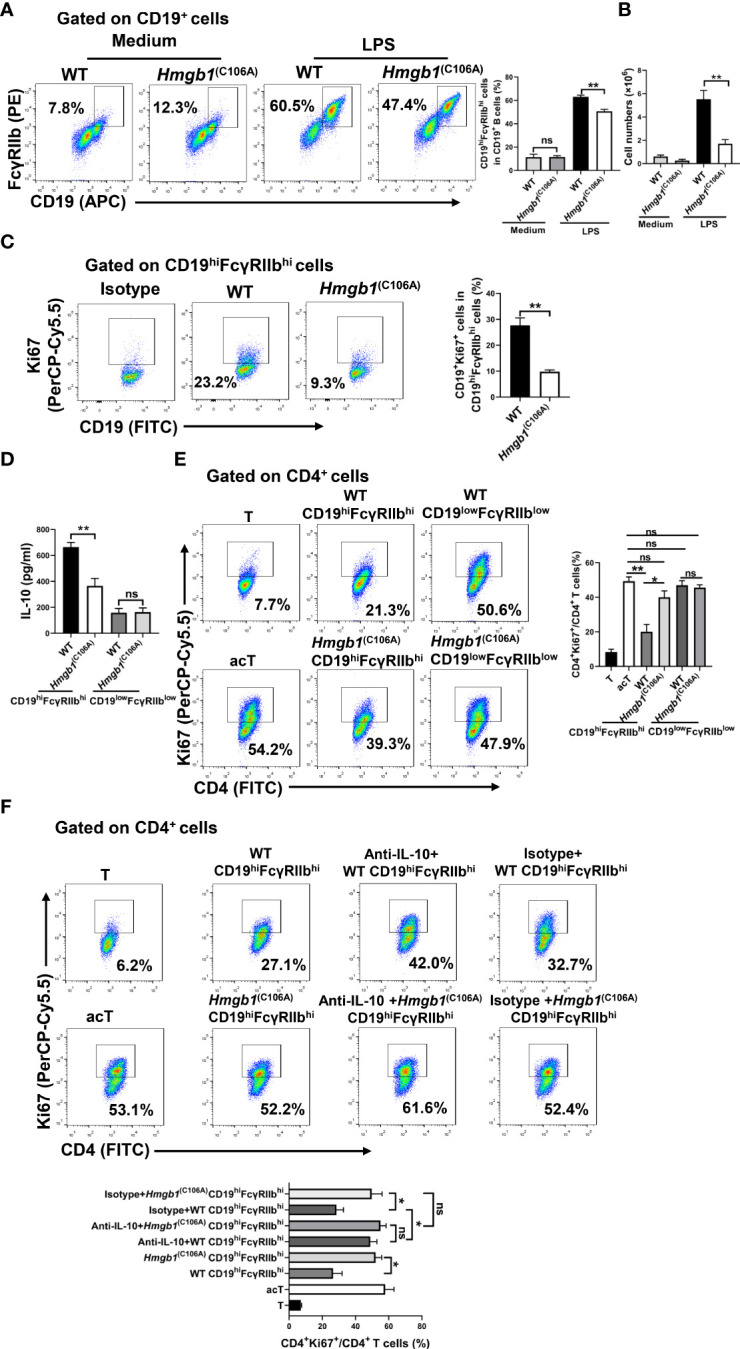
Decreased expansion and suppressive function of CD19^hi^FcγRIIb^hi^ B cells among *Hmgb1* ^(C106A)^ B cells. **(A, B)** Splenic CD19^+^ B cells from *Hmgb1* ^(C106A)^ or WT mice were treated with or without LPS for 2 days. Flow cytometry was performed to assess the percentage **(A)** and number **(B)** of CD19^hi^FcγRIIb^hi^ B cells. Data are shown as means ± SEMs of three independent experiments. **(C)** Highly purified splenic CD19^+^ B cells from *Hmgb1* ^(C106A)^ or WT mice were cocultured with LPS for 48 h *in vitro*, and the percentage of proliferating (Ki67^+^) CD19^hi^FcγRIIb^hi^ B cells among WT and *Hmgb1* ^(C106A)^ B cells was analyzed by flow cytometry with Ki67 staining. Data are shown as means ± SEMs of three independent experiments. **(D)** Splenic CD19^+^ B cells from *Hmgb1* ^(C106A)^ or WT mice were treated with LPS for 2 days; CD19^hi^FcγRIIb^hi^ B cells and CD19^low^FcγRIIb^low^ B cells were sorted from WT and *Hmgb1* ^(C106A)^ B cells by flow cytometry. CD19^hi^FcγRIIb^hi^ B cells and CD19^low^FcγRIIb^low^ B cells (2 × 10^5^) sorted from *Hmgb1* ^(C106A)^ or WT B cells were plated in 96-well plates. After 24 h, supernatants were collected for measurement of IL-10 levels by ELISA. Data are shown as means ± SEMs of three independent experiments. **(E, F)** Splenic CD19^+^ B cells from *Hmgb1* ^(C106A)^ or WT mice were treated with LPS for 2 days; CD19^hi^FcγRIIb^hi^ B cells and CD19^low^FcγRIIb^low^ B cells were sorted from WT and *Hmgb1* ^(C106A)^ B cells by flow cytometry. CD19^hi^FcγRIIb^hi^ B cells (2 × 10^5^) sorted from *Hmgb1* ^(C106A)^ or WT B cells were cocultured with anti-CD3/CD28-activated CD4^+^ T cells (2 × 10^5^) **(E)** in the presence or absence of anti-IL-10 **(F)**. After 3 days, CD4^+^ T cell proliferation was analyzed by flow cytometry. Data are shown as means ± SEMs of three independent experiments. *P < 0.05; **P < 0.01; NS, not significant.

Because HMGB1 is expressed in B cells (24), we examined whether the HMGB1 (C106A) mutation decreased the number and suppressive function of CD19^hi^FcγRIIb^hi^ B cells. Highly purified CD19^+^ B cells from *Hmgb1* ^(C106A)^ or WT mice were cocultured with LPS (10 μg/mL) for 48 h *in vitro*; the number and percentage of CD19^hi^FcγRIIb^hi^ B cells were then determined by flow cytometry. The percentage and number of CD19^hi^FcγRIIb^hi^ B cells among were significantly lower LPS-treated *Hmgb1* ^(C106A)^ B cells than among corresponding WT controls (**Figures 3A**, **B**). Next, highly purified CD19^+^ B cells from *Hmgb1* ^(C106A)^ or WT mice were cocultured with LPS for 48 h *in vitro*; the percentages of proliferating (Ki67^+^) and apoptotic (AnnexinV^+^7AAD^+^) CD19^hi^FcγRIIb^hi^ B cells in WT and *Hmgb1* ^(C106A)^ spleens were analyzed by flow cytometry. The proliferation of CD19^hi^FcγRIIb^hi^ B cells from LPS-treated *Hmgb1* ^(C106A)^ B cells was decreased, compared with those cells from WT mice (**Figure 3C**). However, the rate of apoptosis among CD19^hi^FcγRIIb^hi^ B cells did not significantly differ between LPS-treated WT and *Hmgb1* ^(C106A)^ B cells (**Figure S5**).

We next investigated whether the HMGB1 (C106A) mutation in B cells affected IL-10 secretion and the immunosuppressive function of CD19^hi^FcγRIIb^hi^ B cells. Highly purified CD19^+^ B cells from *Hmgb1* ^(C106A)^ or WT mice were cocultured with LPS for 48 h *in vitro*; CD19^hi^FcγRIIb^hi^ B cells and CD19^low^FcγRIIb^low^ B cells from WT and *Hmgb1* ^(C106A)^ B cells were then sorted by flow cytometry. CD19^hi^FcγRIIb^hi^ B cells or CD19^low^FcγRIIb^low^ B cells (2 × 10^5^) were plated onto 96-well plates. After 24 h, the supernatants were collected for measurement of IL-10 levels by ELISA. Purified CD19^hi^FcγRIIb^hi^ B cells or CD19^low^FcγRIIb^low^ B cells (2 × 10^5^) were cocultured with anti-CD3/CD28-activated CD4^+^ T cells (2 × 10^5^) in the presence or absence of anti-IL-10. CD19^hi^FcγRIIb^hi^ B cells sorted from LPS-treated *Hmgb1* ^(C106A)^ B cells secreted less IL-10, compared with WT B cells (**Figure 3D**). CD19^hi^FcγRIIb^hi^ B cells sorted from LPS-treated *Hmgb1* ^(C106A)^ B cells showed a significantly decreased ability to inhibit CD4^+^ T cell proliferation because of their reduced IL-10 secretion (**Figures 3E**, **F**). Therefore, the HMGB1 (C106A) mutation in B cells reduced the expansion and suppressive function of CD19^hi^FcγRIIb^hi^ B cells.

### HMGB1 promotes the expansion of IL-10-producing CD19^hi^FcγRIIb^hi^ B cells *in vitro*

**Figure 4:**
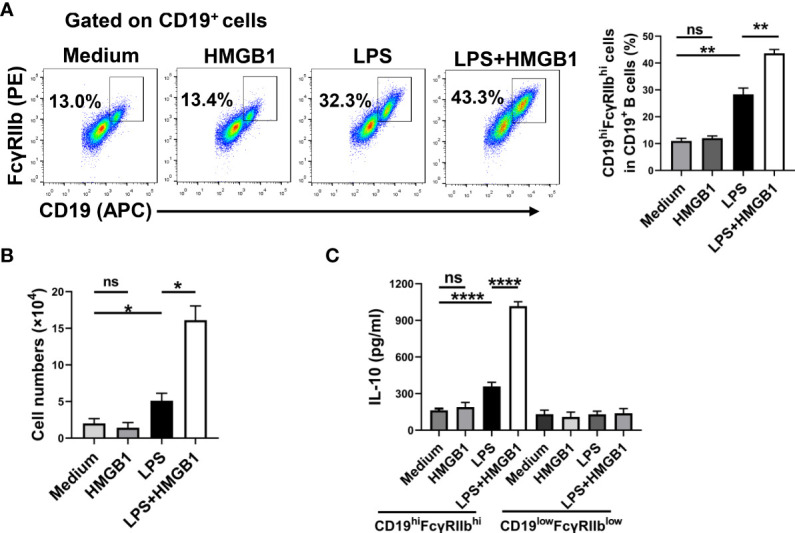
HMGB1 promotes the expansion and IL-10 secretion of CD19^hi^FcγRIIb^hi^ B cells *in vitro*. Sorted CD19^+^B cells (2 × 10^5^/well) from C57BL/6 mice were cultured with HMGB1 (10 μg/mL) and LPS (500 ng/mL) for 2 days. **(A, B)** Flow cytometry was performed to assess the percentage **(A)** and number **(B)** of CD19^hi^FcγRIIb^hi^ B cells. Data are shown as means ± SEMs of three independent experiments. **(C)** CD19^hi^FcγRIIb^hi^ B cells and CD19^low^FcγRIIb^low^ B cells were sorted by flow cytometry. CD19^hi^FcγRIIb^hi^ B cells or CD19^low^FcγRIIb^low^ B cells (2 × 10^5^) were plated on 96-well plates for 24 h. IL-10 secretion by CD19^hi^FcγRIIb^hi^ B cells was analyzed by ELISA. Data are shown as means ± SEMs of four independent experiments. *P < 0.05; **P < 0.01; ****P <0.0001; NS, not significant.

LPS or CpG ODN promotes the expansion of CD19^hi^FcγRIIb^hi^ B cells *in vitro*. To determine whether HMGB1 promotes the expansion of CD19^hi^FcγRIIb^hi^ B cells *in vitro*, we treated purified splenic CD19^+^ B cells with HMGB1(10 μg/mL), LPS (500 ng/mL), or HMGB1 plus LPS. After 48 h, the percentage and number of CD19^hi^FcγRIIb^hi^ B cells were determined by flow cytometry. HMGB1 did not promote the proliferation of CD19^hi^FcγRIIb^hi^ B cells, although it increased CD19^hi^FcγRIIb^hi^ B cell expansion among LPS-activated B cells (**Figures 4A**, **B**). Moreover, HMGB1 enhanced CD19^hi^FcγRIIb^hi^ B cell expansion among CpG ODN-activated B cells (**Figures S6A**, **B**).

Next, we determined whether HMGB1 promotes IL-10 secretion by CD19^hi^FcγRIIb^hi^ B cells. Purified splenic CD19^+^ B cells were treated with HMGB1 (10 μg/mL), LPS (500 ng/mL), or HMGB1 plus LPS. After 48 h, CD19^hi^FcγRIIb^hi^ B cells and CD19^low^FcγRIIb^low^ B cells were sorted by flow cytometry. CD19^hi^FcγRIIb^hi^ B cells or CD19^low^FcγRIIb^low^ B cells (2 × 10^5^) were plated in 96-well plates for 24 h. Supernatants were collected and IL-10 levels were evaluated by ELISA. HMGB1 promoted IL-10 secretion by LPS-activated CD19^hi^FcγRIIb^hi^ B cells (**Figure 4C**). Moreover, HMGB1 promoted IL-10 secretion by CpG ODN-activated CD19^hi^FcγRIIb^hi^ B cells (**Figure S6C**). Therefore, HMGB1 promotes the expansion and IL-10 secretion of CD19^hi^FcγRIIb^hi^ B cells *in vitro*.

### The HMGB1 (C106A) mutation inhibits CD19^hi^FcγRIIb^hi^ B cell expansion and function *via* STAT3

**Figure 5:**
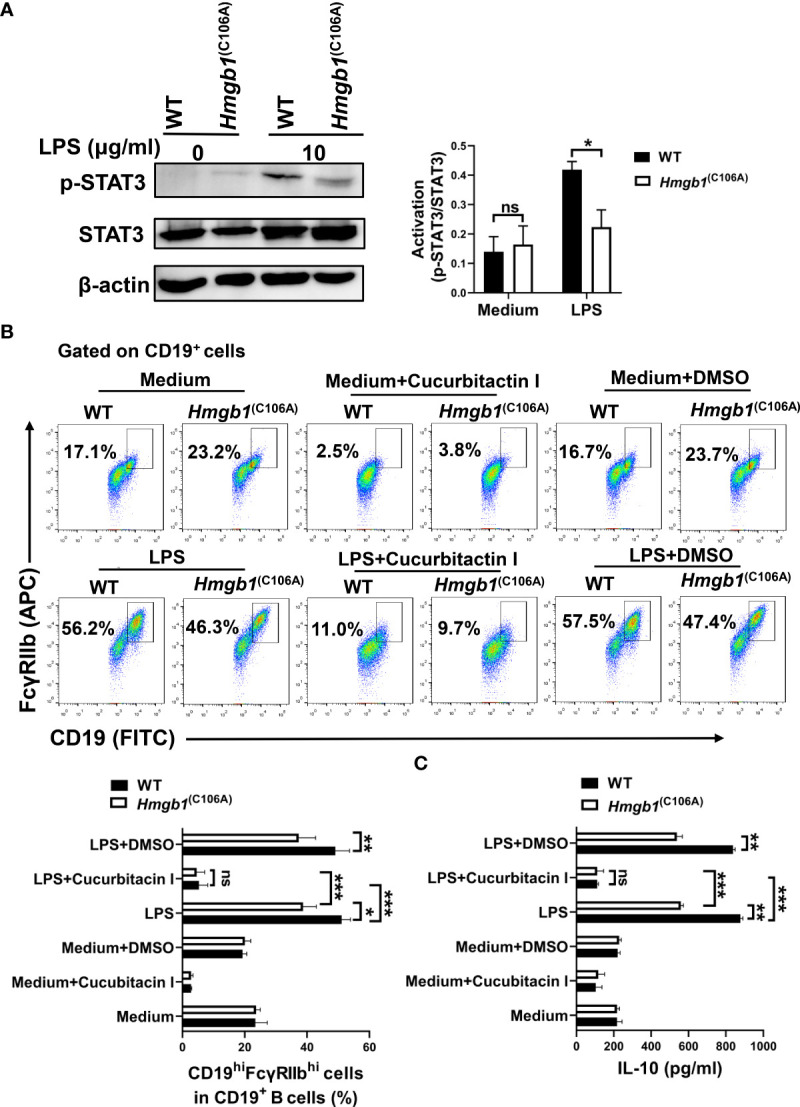
*Hmgb1* ^(C106A)^ inhibits STAT3-mediated expansion and function of CD19^hi^FcγRIIb^hi^ B cells. **(A)** Splenic CD19^+^B cells sorted from *Hmgb1* ^(C106A)^ or WT mice were cultured with or without LPS. After 2 days, total proteins were extracted from B cells, and STAT3 phosphorylation was determined by Western blotting. Data are shown as means ± SEMs of three independent experiments. **(B)** CD19^+^B cells (2 × 10^5^/well) sorted from *Hmgb1* ^(C106A)^ or WT mice were treated with LPS in the presence or absence of cucurbitacin I or dimethyl sulfoxide in 96-well plates. After 2 days, B cells were collected and subjected to flow cytometry to assess the percentage of CD19^hi^FcγRIIb^hi^ B cells. Data are shown as means ± SEMs of three independent experiments. **(C)** CD19^+^B cells (2 × 10^5^/well) sorted from *Hmgb1* ^(C106A)^ or WT mice were treated with LPS in the presence or absence of cucurbitacin I or dimethyl sulfoxide in 96-well plates. After 2 days, supernatants were collected for measurement of IL-10 levels by ELISA. Data are shown as means ± SEMs of three independent experiments. *P < 0.05; **P < 0.01; ***P < 0.001; NS, not significant.

HMGB1 mediates JAK2/STAT3 signaling way (25), and STAT3 is implicated in Breg differentiation and function (26). We evaluated the effect of the HMGB1 (C106A) mutation in B cells on STAT3 phosphorylation. CD19^+^ B cells from WT and *Hmgb1* ^(C106A)^ mice were stimulated with or without LPS. After 48 h, proteins were extracted from LPS-activated WT and *Hmgb1* ^(C106A)^ B cells; STAT3 phosphorylation was evaluated by Western blotting. Although the levels of phosphorylated STAT3 (S727) were similar in WT and *Hmgb1* ^(C106A)^ B cells without LPS stimulation, they were significantly lower in LPS-treated *Hmgb1* ^(C106A)^ B cells than in the corresponding WT B cells (**Figure 5A**).

Splenic CD19^+^ B cells sorted from *Hmgb1* ^(C106A)^ or WT mice were pretreated with cucurbitacin I (an inhibitor of STAT3) for 1 h, followed by LPS for 48 h. Cells were collected and the percentage of CD19^hi^FcγRIIb^hi^ B cells was determined by flow cytometry. When STAT3 activity was inhibited, the percentage of CD19^hi^FcγRIIb^hi^ B cells was decreased in both LPS-activated WT and *Hmgb1* ^(C106A)^ B cells; however, the percentage of CD19^hi^FcγRIIb^hi^ B cells did not significantly differ between LPS-activated WT and *Hmgb1* ^(C106A)^ B cells (**Figure 5B**). Furthermore, when STAT3 activity was inhibited, IL-10 secretion by LPS-activated WT and *Hmgb1* ^(C106A)^ B cells was decreased; there was no significant difference in IL-10 secretion between LPS-activated WT and *Hmgb1* ^(C106A)^ B cells (**Figure 5C**). Therefore, the HMGB1 (C106A) mutation in B cells suppressed STAT3 activation, thus inhibiting the expansion and IL-10 secretion of CD19^hi^FcγRIIb^hi^ B cells.

### Increased percentages of CD19^hi^FcγRIIb^hi^ B cells in peripheral blood from patients with sepsis

**Figure 6:**
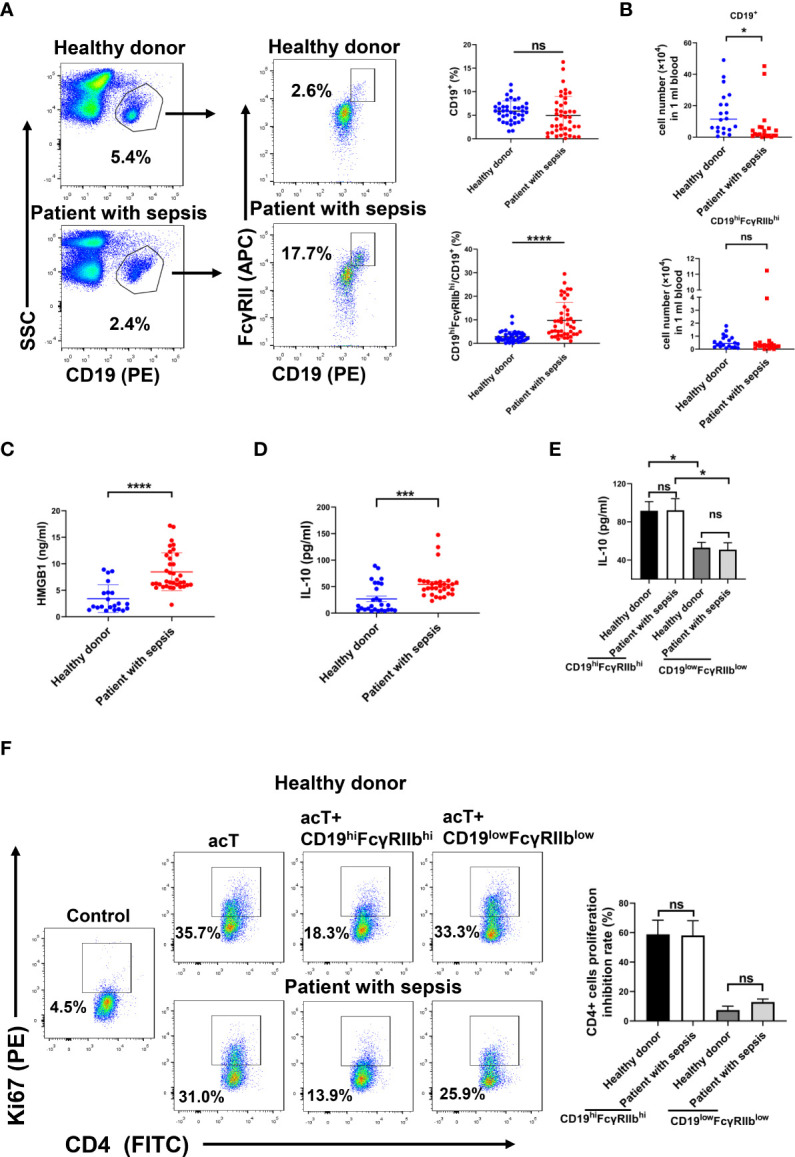
Increased percentage of CD19^hi^FcγRIIb^hi^ B cells in peripheral blood from patients with sepsis. **(A)** Percentages of B cells and CD19^hi^FcγRIIb^hi^ B cells among PBMCs of patients with sepsis and healthy donors were analyzed by flow cytometry. Data are shown as means ± SEMs; n = 40 samples for healthy donors, n = 44 samples for patients with sepsis. **(B)** Numbers of CD19^+^ B cells and CD19^hi^FcγRIIb^hi^ B cells among PBMCs analyzed by flow cytometry. Data are shown as means ± SEMs; n = 20 samples per group. **(C)** HMGB1 levels in plasma analyzed by ELISA. Data are shown as means ± SEMs; n = 21 samples for healthy donors, n = 36 samples for patients with sepsis. **(D)** IL-10 levels in plasma analyzed by ELISA. Data are shown as means ± SEMs; n = 27 samples for healthy donors, n = 33 samples for patients with sepsis. **(E)** CD19^hi^FcγRIIb^hi^ B cells and CD19^low^FcγRIIb^low^ B cells were sorted from PBMCs of patients with sepsis and healthy donors by flow cytometry. CD19^hi^FcγRIIb^hi^ B cells or CD19^low^FcγRIIb^low^ B cells (2 × 10^5^) were plated in 96-well plates. After 24 h, supernatants were collected for measurement of IL-10 levels by ELISA. Data are shown as means ± SEMs of three representative experiments; n = 3 samples per group. **(F)** CD19^hi^FcγRIIb^hi^ B cells (1 × 10^5^/well) were cocultured with autologous anti-CD3/CD28 activated CD4^+^ T cells (1 × 10^5^/well) for 2 days, and Ki67 expression in CD4^+^ T cells was analyzed by flow cytometry. Results are representative of three independent experiments. Data are shown as means ± SEMs of three representative experiments; n = 3 samples per group. *P < 0.05; ***P < 0.001; ****P < 0.0001; NS, not significant.

CD19^hi^FcγRIIb^hi^ B cells are present in human peripheral blood, where they may inhibit autologous CD4^+^ T cell proliferation. We used flow cytometry to examine whether CD19^hi^FcγRIIb^hi^ B cells were increased in patients with sepsis. The percentage of B cells among PBMCs did not differ between patients with sepsis and healthy donors, while the percentages of CD19^hi^FcγRIIb^hi^ B cells were significantly greater in patients with sepsis than in healthy donors (**Figure 6A**). Moreover, the number of B cells was smaller in patients with sepsis than in healthy donors, but the numbers of CD19^hi^FcγRIIb^hi^ B cells did not differ between groups (**Figure 6B**).

ELISA showed that the HMGB1 and IL-10 levels were higher in patients with sepsis than in healthy donors (**Figures 6C**, **D**). We next investigated whether CD19^hi^FcγRIIb^hi^ B cells have greater suppressive activity in patients with sepsis than in healthy donors. CD19^hi^FcγRIIb^hi^ B cells and CD19^low^FcγRIIb^low^ B cells were sorted from PBMCs of patients with sepsis and healthy donors. CD19^hi^FcγRIIb^hi^ B cells or CD19^low^FcγRIIb^low^ B cells (2 × 10^5^) were plated in 96-well plates for 24 h; IL-10 levels in supernatant were then evaluated. CD19^hi^FcγRIIb^hi^ B cells secreted more IL-10, compared with CD19^low^FcγRIIb^low^ B cells, from both patients with sepsis and healthy donors. However, there was no difference in IL-10 secretion by CD19^hi^FcγRIIb^hi^ B cells between patients with sepsis and healthy donors (**Figure 6E**). CD19^hi^FcγRIIb^hi^ B cells, CD19^low^FcγRIIb^low^ B cells, and CD4^+^ T cells were sorted from PBMCs of patients with sepsis and healthy donors. Purified CD19^hi^FcγRIIb^hi^ B cells or CD19^low^FcγRIIb^low^ B cells (1 × 10^5^) were cocultured with anti-CD3/CD28-activated CD4^+^ T cells (1 × 10^5^) for 2 days. CD19^hi^FcγRIIb^hi^ B cells suppressed autologous CD4^+^ T cell proliferation. However, there was no difference in the suppressive activity of CD19^hi^FcγRIIb^hi^ B cells between patients with sepsis and healthy donors (**Figure 6F**). Therefore, compared with healthy donors, the percentages of CD19^hi^FcγRIIb^hi^ B cells in patients with sepsis were increased, but the suppressive function of CD19^hi^FcγRIIb^hi^ B cells did not significantly differ.

## Discussion

Bregs lack a specific transcription factor and secrete high levels of both IL-10 and TGF-β (27). Our previous studies have showed that there is a new subset of IL-10-producing regulatory B cells with high CD19 and FcγRIIb expression in mice (10). The percentages of CD19^hi^FcγRIIb^hi^ B cells are increased in the spleens of mice with LPS-induced inflammation, but the expansion and function of CD19^hi^FcγRIIb^hi^ B cells during inflammation are unclear. HMGB1 is a damage-associated molecular pattern protein that is released from the nucleus to the extracellular space during inflammation and infection (11, 28). HMGB1 regulates various immunosuppressive cell types; for example, it enhances the immunosuppressive activity of Tregs (17)and induces the differentiation of bone marrow cells to myeloid-derived suppressor cells (18). However, it has been unclear whether HMGB1 regulates CD19^hi^FcγRIIb^hi^ B cell expansion and function. Therefore, we investigated the potential regulation of mouse spleen CD19^hi^FcγRIIb^hi^ B cells by HMGB1 during inflammation.

The percentage of CD19^hi^FcγRIIb^hi^ B cells from mice with LPS-induced inflammation was significantly increased, compared with the percentage of such cells from WT mice; the expansion of CD19^hi^FcγRIIb^hi^ B cells in the spleen of *Hmgb1* ^(C106A)^ mice with LPS-induced inflammation was significantly decreased. CD19^hi^FcγRIIb^hi^ B cells from the spleen of *Hmgb1* ^(C106A)^ mice with LPS-induced inflammation secreted less IL-10. The ability of CD19^hi^FcγRIIb^hi^ B cells from *Hmgb1* ^(C106A)^ mice with LPS-induced inflammation to inhibit the proliferation of activated CD4^+^ T cells was significantly decreased, compared with that ability in cells from WT mice. In addition, we examined the effect of CD19^hi^FcγRIIb^hi^ B cells with the HMGB1 (C106A) mutation on the progression of sepsis. *Hmgb1* ^(C106A)^ significantly reduced the anti-inflammatory effect of CD19^hi^FcγRIIb^hi^ B cells. HMGB1 is expressed in B cells; thus, we explored whether *Hmgb1* ^(C106A)^ in B cells would decrease the percentage, number, and suppressive function of CD19^hi^FcγRIIb^hi^ B cells. *Hmgb1* ^(C106A)^ in B cells reduced the expansion and suppressive function of CD19^hi^FcγRIIb^hi^ B cells.

LPS and CpG ODN induce IL-10 production by CD19^hi^FcγRIIb^hi^ B cells *in vitro*. The B-cell receptor, CD40L, and Toll-like receptor (TLR) pathways are required for the induction and development of IL-10-producing Bregs (29). The main receptors of HMGB1 are TLRs such as TLR2, TLR4, and TLR9 (30). Therefore, we explored whether recombinant HMGB1 could promote the expansion of CD19^hi^FcγRIIb^hi^ B cells *in vitro*. HMGB1 alone did not induce CD19^hi^FcγRIIb^hi^ B cells, while it enhanced the expansion of CD19^hi^FcγRIIb^hi^ B cells among LPS- or CpG ODN-activated B cells. Therefore, in contrast to LPS and CpG ODN, HMGB1 does not induce CD19^hi^FcγRIIb^hi^ B cells, possibly because of the activation of different signaling pathways. The underlying mechanism warrants further investigation.

The mechanisms underlying Bregs differentiation are unclear but involve transcription factors such as IRF4 (31), STAT3 (26), and c-MAF (32). Moreover, extracellular HMGB1 activates the STAT3 signaling pathway (33) and intracellular HMGB1 binds to STAT3 (34). Therefore, we tested whether *Hmgb1* ^(C106A)^ affects the expansion of CD19^hi^FcγRIIb^hi^ B cells *via* the STAT3 signaling pathway. p-STAT3 expression was significantly reduced in LPS-activated *Hmgb1* ^(C106A)^ B cells, compared with LPS-activated WT B cells. The inhibition of STAT3 activity decreased the percentages and IL-10 secretion of CD19^hi^FcγRIIb^hi^ B cells in both LPS-activated WT and *Hmgb1* ^(C106A)^ B cells. The percentage and IL-10 secretion of CD19^hi^FcγRIIb^hi^ B cells did not significantly differ between LPS-activated WT and *Hmgb1* ^(C106A)^ B cells. Therefore, *Hmgb1* ^(C106A)^ inhibits the expansion and function of CD19^hi^FcγRIIb^hi^ B cells in a STAT3-dependent manner.

Sepsis is an overreaction to a pathogen and affects most types of immune cells (35, 36). In an immunosuppressed state, the numbers of lymphocytes are greatly reduced and the numbers of negative regulatory cells are increased (37, 38). Patients who survive sepsis are typically in a state of prolonged immunosuppression and likely to experience nosocomial infection (39). Here, we found that the percentage of CD19^hi^FcγRIIb^hi^ B cells was significantly increased in patients who survived sepsis. The HMGB1 content in plasma was higher in patients with sepsis than in healthy donors. It has been reported that some inflammatory cytokines, such as IL-6 and IL-1β, can induce IL-10-producing regulatory B cells generation. However, whether these cytokines, together with HMGB1, also affect the frequency of CD19^hi^FcγRIIb^hi^ B cells in peripheral blood of patients with sepsis needs to be investigated further. There was no significant difference in the function of CD19^hi^FcγRIIb^hi^ B cells between healthy donors and patients with sepsis. Mouse B cells express TLR2 and TLR4. Human B cells have been shown to express TLR1, TLR6 and TLR7, but exhibit minimal or no TLR2, TLR3, TLR4 and TLR5 expression (40). TLRs activation plays an important role in the development and function of regulatory B cells expressing IL-10 (41). The difference of TLRs expression on human B cells and mouse B cells may explain why exogenous HMGB1 enhanced the suppressive function of mouse CD19^hi^FcγRIIb^hi^ B cells but not human CD19^hi^FcγRIIb^hi^ B cells. Future studies should investigate whether the frequency of CD19^hi^FcγRIIb^hi^ B cells is related to disease stage and prognosis in patients with sepsis.

In conclusion, the HMGB1 (C106A) mutation in B cells reduced the expansion and suppressive function of CD19^hi^FcγRIIb^hi^ B cells by blocking STAT3 phosphorylation. HMGB1 promoted the expansion of CD19^hi^FcγRIIb^hi^ B cells among LPS or CpG ODN-activated B cells. Moreover, the percentage of CD19^hi^FcγRIIb^hi^ B cells was significantly increased in patients with sepsis.

## Data availability statement

The raw data supporting the conclusions of this article will be made available by the authors, without undue reservation.

## Ethics statement

The studies involving human participants were reviewed and approved by The Ethics Committee of the Medical College of Yangzhou University. The patients/participants provided their written informed consent to participate in this study. The animal study was reviewed and approved by The Ethics Committee of the Medical College of Yangzhou University.

## Author contributions

ML, JZ, RY, HY and YD performed the experiments in this study. LQ and FM designed the experiments, analyzed data, and wrote the paper. All authors contributed to the article and approved the submitted version.

## Funding

This work was supported by the National Natural Science Foundation of China (81771689, 81373130 and 81001308), the Six Talent Peak Projects in Jiangsu Province (YY-050), Natural Science Foundation of Jiangsu Province for Distinguished Young Scholars (BK20200004) and the Young Academic Leaders Project of Yangzhou University (201714).

## Conflict of interest

The authors declare that the research was conducted in the absence of any commercial or financial relationships that could be construed as a potential conflict of interest.

## Publisher’s note

All claims expressed in this article are solely those of the authors and do not necessarily represent those of their affiliated organizations, or those of the publisher, the editors and the reviewers. Any product that may be evaluated in this article, or claim that may be made by its manufacturer, is not guaranteed or endorsed by the publisher.
