# Supplementary material for: The HMGB1 (C106A) mutation inhibits IL-10-producing CD19hiFcγRIIbhi B cell expansion by suppressing STAT3 activation in mice
Source: Front Immunol. 2022 Aug 2;13:975551. doi: 10.3389/fimmu.2022.975551 (PMC9378787; doi:10.3389/fimmu.2022.975551)
Supplement: Supplementary file 1 [file DataSheet_1.docx]

**Table S1. Characteristics of the study population (N=44).**

| Variable | Patient with sepsis (N=44) |
| --- | --- |
| Age (years old) | 68.20±15.90 |
| Gender (Male/ Female) | 28/16 |
| WBC (× 10^9^) | 12.40±7.53 |
| PCT (ng/ml) | 5.72±11.18 |
| NE (%) | 80.89±14.01 |

WBC , white blood cell count ; NE , neutrophil ; PCT , procalcitonin ;

**Table S2. Key resources**

| Reagent or resource | Source | Identifier |
| --- | --- | --- |
| Antibodies |  |  |
| CD4 MicroBeads mouse | Miltenyi Biotec | 130-117-043 |
| CD19 MicroBeads mouse | Miltenyi Biotec | 130-052-201 |
| FITC anti-mouse CD19 (clone 6D5) | BioLegend | 115506 |
| APC anti-mouse CD19 (clone 1D3) | eBioscience | 17-0193-82 |
| APC anti-mouse CD32b (clone AT130-2) | eBioscience | 17-0321-82 |
| PE anti-mouse CD32b (clone AT130-2) | eBioscience | 12-0321-82 |
| APC anti-mouse CD3 (clone17a2) | BioLegend | 100236 |
| PE anti-mouse NK-1.1 (clone PK136) | BioLegend | 108708 |
| FITC anti-mouse CD8a (clone 53-6.7) | eBioscience | 11-0081-82 |
| FITC anti-mouse CD4 (clone GK1.5) | eBioscience | 11-0041-82 |
| FITC anti-mouse CD11b (clone M1/70) | eBioscience | 11-0112-85 |
| APC anti-mouse F4/80 (clone BM8) | BioLegend | 123116 |
| PE-Cy7 anti-mouse Ly-6G/Ly-6C (clone RB6-8C5) | eBioscience | 25-5931-82 |
| APC anti-mouse CD25 (clone PC61) | BioLegend | 102012 |
| PE anti-mouse Foxp3 (clone NRPF-30) | eBioscience | 12-4771-82 |
| FITC annexin V | MULTI SCIENCES | AP101-30-kit |
| PerCP/Cy5.5 anti-mouse Ki67 (clone 16A8) | BioLegend | 652424 |
| PE anti-human CD19 (clone SJ25C1) | MULTI SCIENCES | AH0190204-100 |
| FITC anti-human CD19 (clone HIB19) | eBioscience | 11-0199-42 |
| PerCP/Cy5.5 anti-human CD19 (clone HIB19) | eBioscience | 45-0199-42 |
| APC anti-human CD32 (clone 6C4) | eBioscience | 17-0329-42 |
| PE anti-human Ki67 (clone Ki-67) | BioLegend | 350503 |
| FITC anti-human CD4 (clone RPA-T4) | MULTI SCIENCES | AH00401-100 |
| Goat anti-rabbit IgG (H+L) HRP | Cell Signaling Technology | 7074P2 |
| p-STAT3 (S727) Rabbit Ab | Cell Signaling Technology | 9134T |
| STAT3 (7907) Rabbit mAb | Cell Signaling Technology | 4904T |
| β-actin Monolonal Antibody | Immuno Way | YM3028 |
| Goat anti-mouse IgG (H+L) HRP | GenScript | A00106 |
| PerCP/Cy5.5 rat IgG2a, κ isotype control antibody | BioLegend | 400531 |
| Anti-Mo IL-10 Functionla Grade | eBioscience | 16-7102-81 |
| Normal Goat IgG Control | R&D Systems | AB-108-C |
| Ultra-LEAF Purified anti-mouse CD3ε | BioLegend | 100340 |
| Ultra-LEAF Purified anti-mouse CD28 | BioLegend | 102116 |
| Anti-Human CD28, Functional Grade (Clone:CD28.2) | MULTI SCIENCES | AH028-100 |
| Anti-Human CD3, Functional Grade (Clone: OKT3) | MULTI SCIENCES | AH003-100 |
| Chemicals, peptides, and recombinant proteins |  |  |
| LPS (E.coli O111:B4) | Sigma | L4391-1MG |
| LPS (E.coli O55:B5) | Sigma | L2880-25MG |
| CpG-ODN (5’TCCATGACGTTCCTGATGCT) | Sangon Biotech |  |
| Recombinant Human HMGB1 Protein | R&D Systems | 1690-HGB-050 |
| 7AAD | KeyGEN BioTECH | KGA219 |
| Murine IL-10 | PEPRO TECH | 210-10-10UG |
| Critical commercial assays |  |  |
| Whole Cell Lysis Assay | KeyGen | KGP250 |
| Mouse TGF-β1 ELISA kit | DRKEWE | DKW12-2710-048 |
| Mouse IL-10 ELISA kit | R&D Systems | M1000B |
| Mouse IL-10 ELISA kit | DRKEWE | 1211002 |
| Human IL-10 ELISA kit | MULTI SCIENCES | EK100HS-96 |
| Human HMGB1 ELISA kit | Solarbio | SEKH-0409 |
| Software and algorithms |  |  |
| Prism 8 | GraphPad Software |  |
| Flow Jo 10 | BD |  |
