# Supplementary material for: The HMGB1 (C106A) mutation inhibits IL-10-producing CD19hiFcγRIIbhi B cell expansion by suppressing STAT3 activation in mice
Source: Front Immunol. 2022 Aug 2;13:975551. doi: 10.3389/fimmu.2022.975551 (PMC9378787; doi:10.3389/fimmu.2022.975551)

**Supplemental Material**

**Figure S1.** **Percentages of T cells, B cells, NK cells, Tregs, and macrophages in the spleen did not differ between WT and *Hmgb1*^(C106A)^ with or without LPS-induced inflammation.** PBS or LPS (10 mg/kg) was injected into WT and *Hmgb1*^(C106A)^ mice. After 7 days, the percentages of immune cells in the spleen were determined by flow cytometry. T cells were CD3^+^CD4^+^ and CD3^+^CD8^+^, macrophages were CD11b^+^F4/80^+^, B cells were CD19^+^, Tregs were CD4^+^CD25^+^Foxp3^+^, and NK cells were CD3^−^NK1.1^+^. Data are shown as means ± SEMs of three independent experiments. NS, not significant.


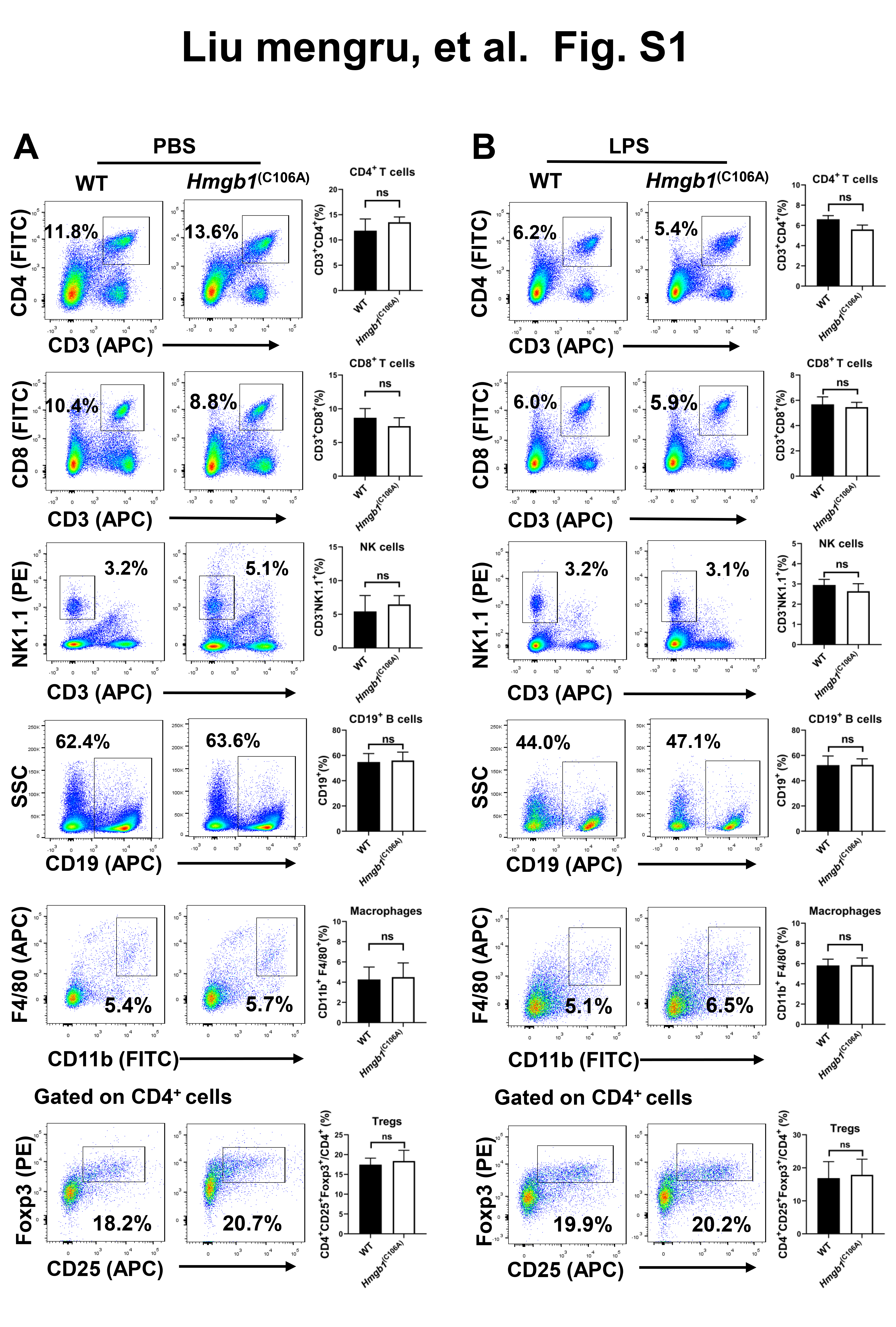


**Figure S2.** **CD19^hi^FcγRIIb^hi^ B cell percentages in lymph nodes were similar in WT and *Hmgb1*^(C106A)^ mice.** LPS (10 mg/kg) was injected into WT and *Hmgb1*^(C106A)^ mice. After 7 days, the percentages of CD19^hi^FcγRIIb^hi^ B cells in lymph nodes of mice with LPS-induced inflammation were analyzed by flow cytometry. Data are shown as means ± SEMs of three independent experiments. NS, not significant.


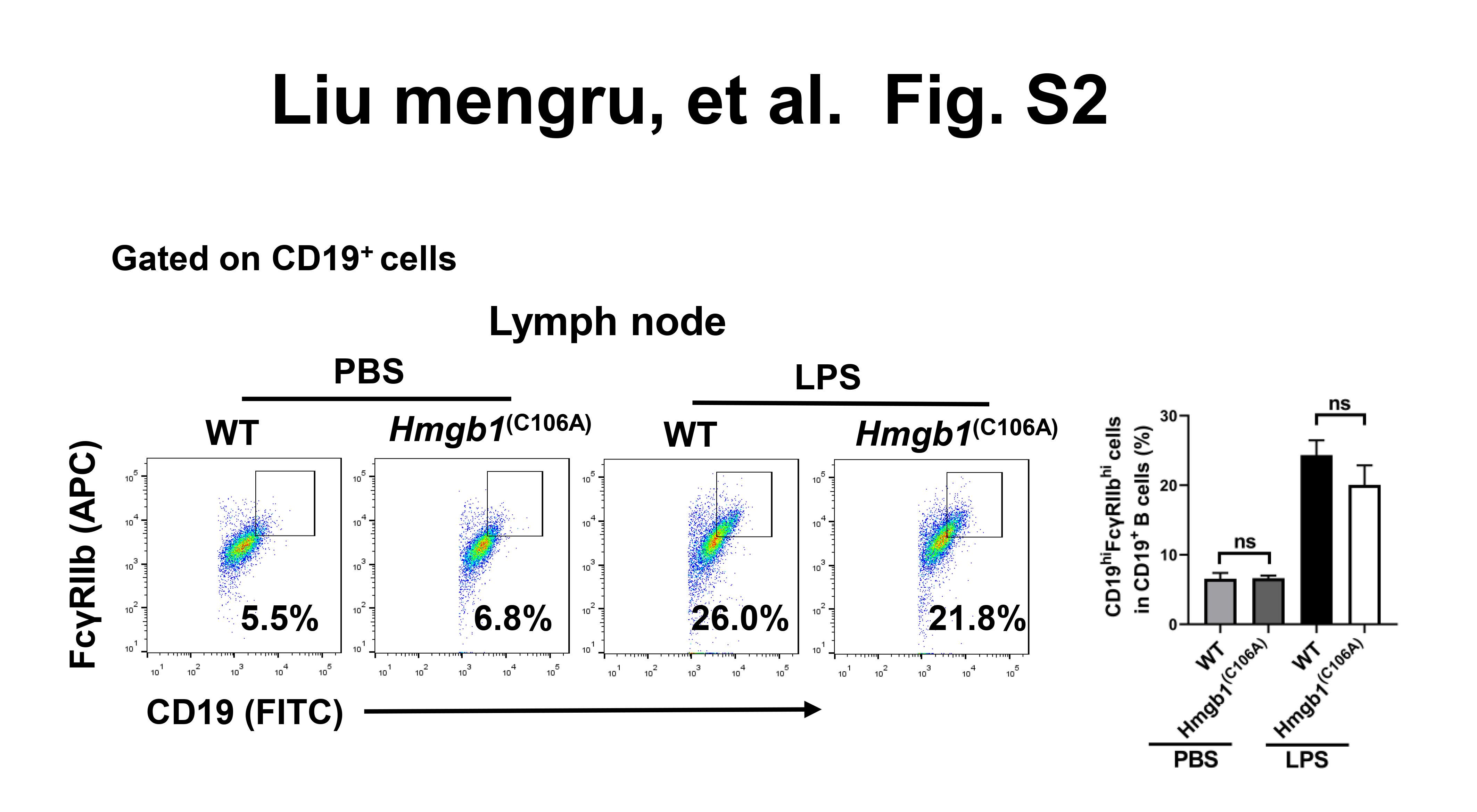


**Figure S3.** **CD19^hi^FcγRIIb^hi^ B cell percentage was decreased in the spleen of *Hmgb1*^(C106A)^ mice with CpG ODN-induced inflammation.** WT and *Hmgb1*^(C106A)^ mice were pre-inoculated with D-GalN (600 mg/kg) for 1 h, then injected with CpG ODN (10 nmol/mouse). After 7 days, the percentages of CD19^hi^FcγRIIb^hi^ B cells in the spleens of mice with CpG ODN-induced inflammation were analyzed by flow cytometry. Data are shown as means ± SEMs of three independent experiments. *** P < 0.001; NS, not significant.

**
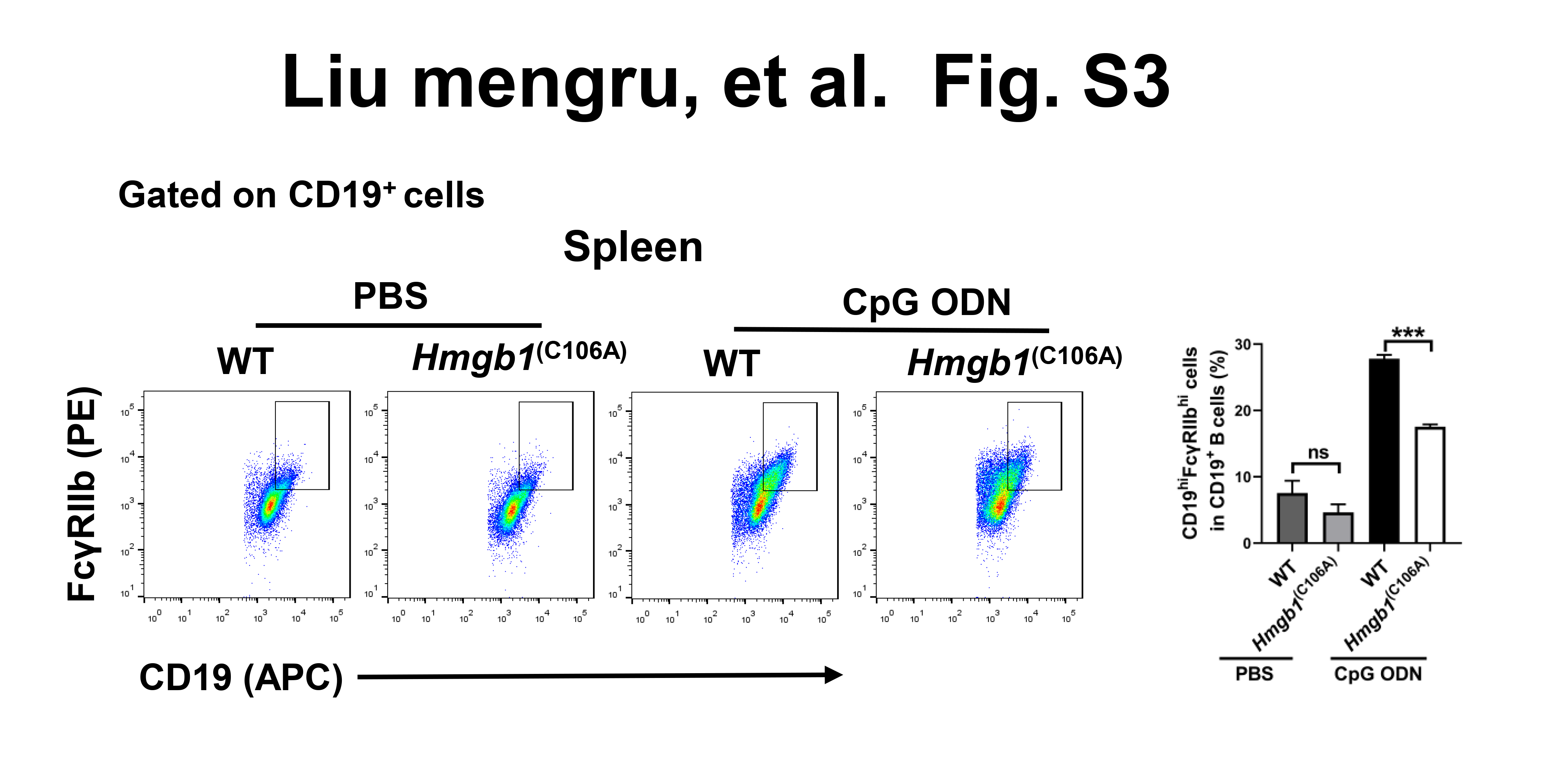
**

**Figure S4. TGF-β secretion by CD19^hi^FcγRIIb^hi^ B cells did not differ between WT and *Hmgb1*^(C106A)^ mice with LPS-induced inflammation.** WT and *Hmgb1*^(C106A)^ mice were injected with LPS (10 mg/kg); splenic CD19^hi^FcγRIIb^hi^ B cells and CD19^low^FcγRIIb^low^ B cells were sorted by flow cytometry on day 7. CD19^hi^FcγRIIb^hi^ B cells or CD19^low^FcγRIIb^low^ B cells (2 × 10^5^) were plated in 96-well plates for 24 h, and TGF-β levels in supernatants were determined by ELISA. Data are shown as means ± SEMs of three independent experiments. NS, not significant.


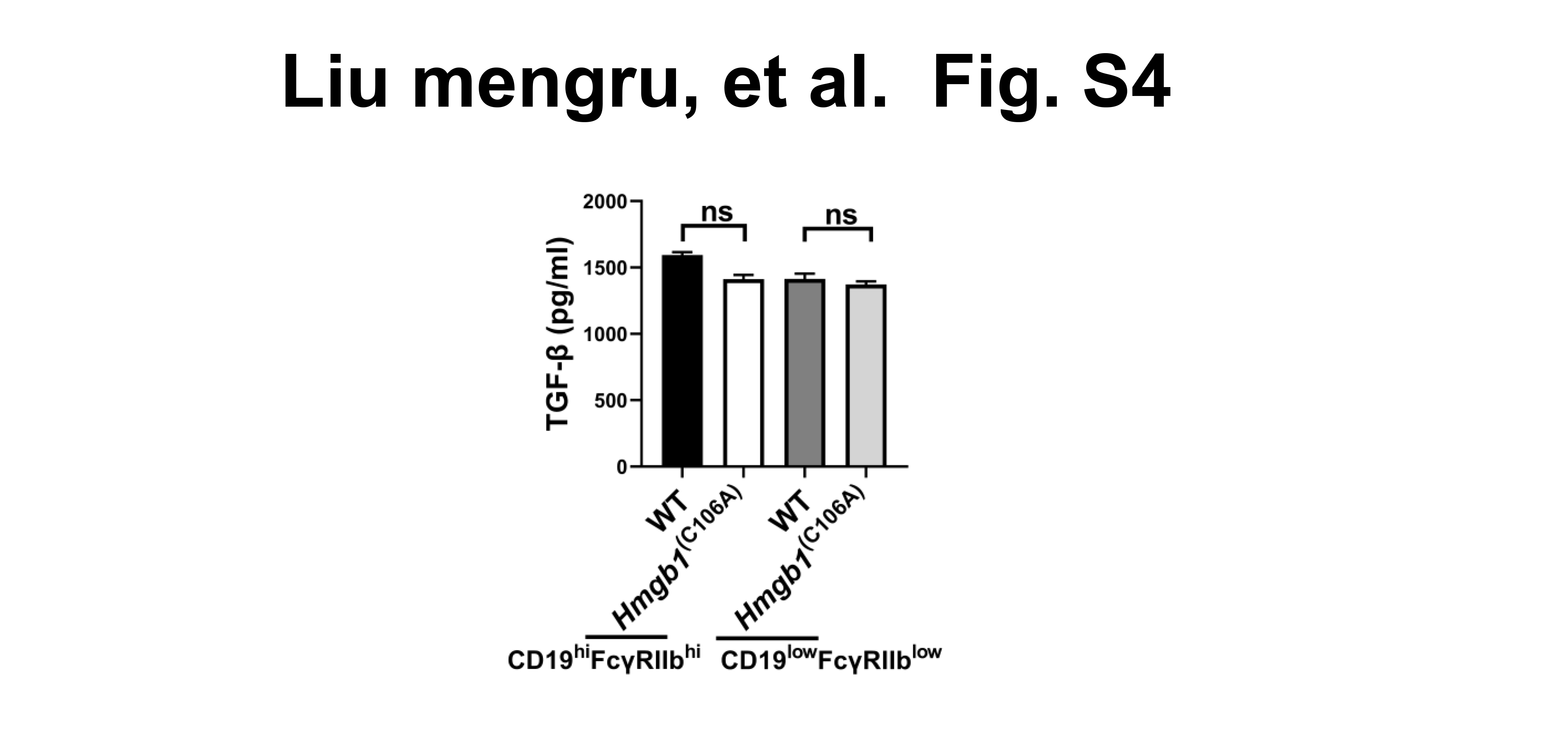


**Figure S5. CD19^hi^FcγRIIb^hi^ B cell apoptosis did not differ between LPS-treated WT and *Hmgb1*^(C106A)^ mice.** CD19^+^ B cells from *Hmgb1*^(C106A)^ or WT mice were cocultured with LPS (10 μg/mL) for 48 h *in vitro*, and the percentage of AnnexinV^+^7AAD^+^ cells in gated CD19^hi^FcγRIIb^hi^ B cells was analyzed by flow cytometry. Data are shown as means ± SEMs of three independent experiments. NS, not significant.


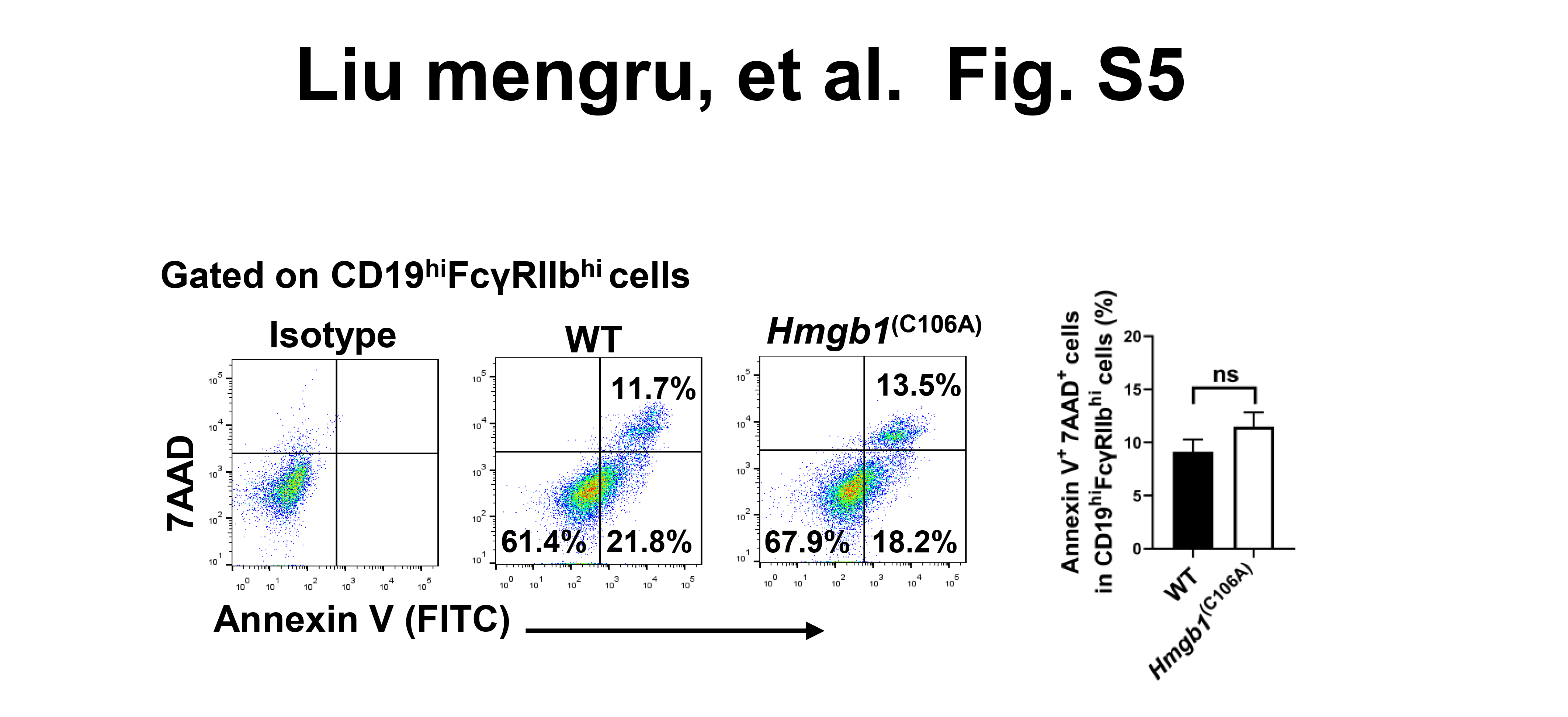


**Figure S6. HMGB1 promotes expansion of IL-10-producing CD19^hi^FcγRIIb^hi^ B cells among CpG ODN-activated B cells *in vitro*.** CD19^+^B cells (2 × 10^5^/well) from C57BL/6 mice were cultured with HMGB1 (10 μg/mL) and CpG ODN (0.02 μM) for 2 days. (A) Flow cytometry was performed to assess the percentage of CD19^hi^FcγRIIb^hi^ B cells. Data are shown as means ± SEMs of three independent experiments. (B) Flow cytometry was performed to assess the number of CD19^hi^FcγRIIb^hi^ B cells. Data are shown as means ± SEMs of four independent experiments. (C) CD19^hi^FcγRIIb^hi^ B cells and CD19^low^FcγRIIb^low^ B cells were sorted by flow cytometry. CD19^hi^FcγRIIb^hi^ B cells or CD19^low^FcγRIIb^low^ B cells (2 × 10^5^) were plated in 96-well plates for 24 h. IL-10 secretion by CD19^hi^FcγRIIb^hi^ B cells was analyzed by ELISA. Data are shown as means ± SEMs of four independent experiments. * P < 0.05; ** P < 0.01; *** P < 0.001; NS, not significant.


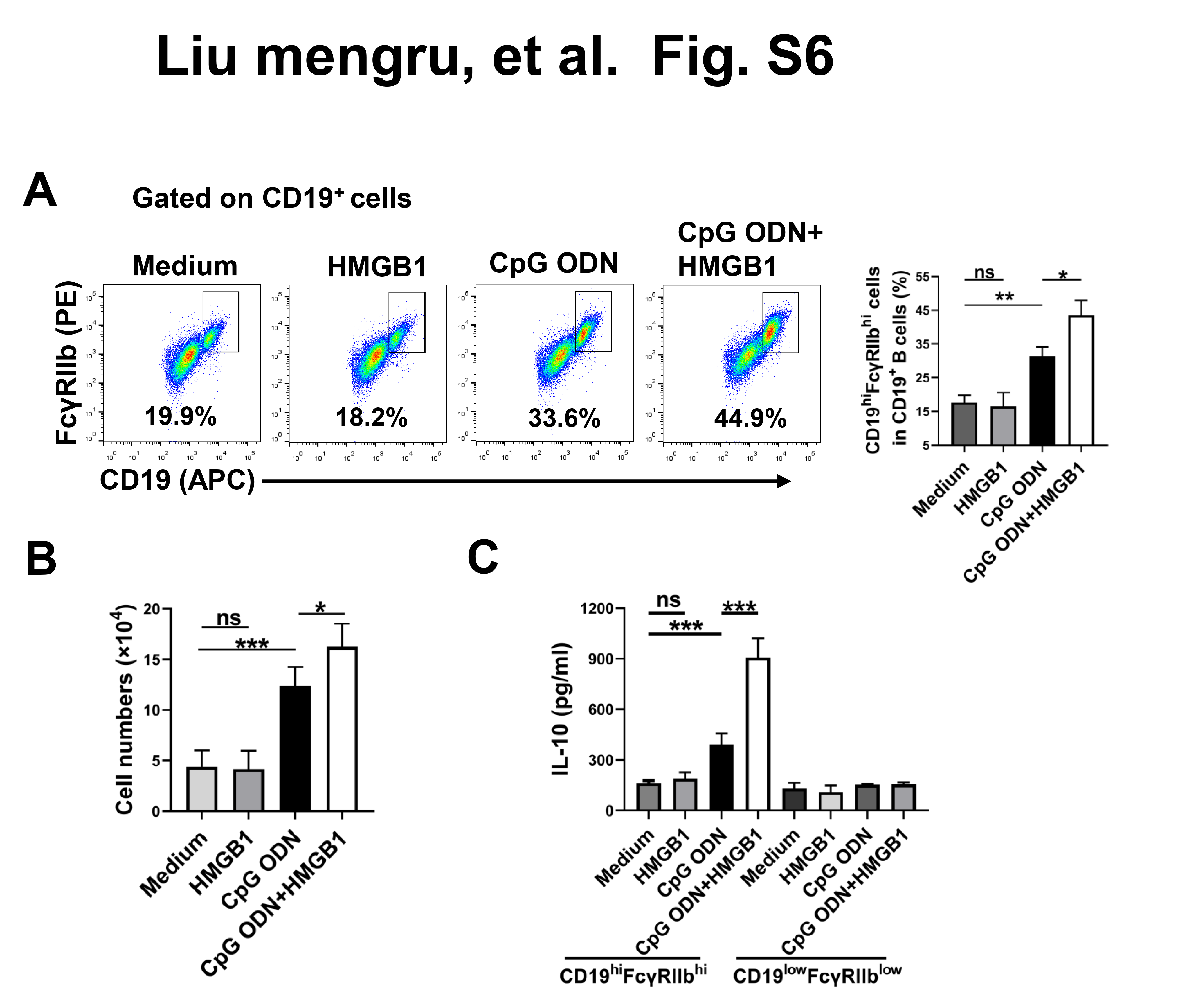

Supplement: Supplementary file 2 [file DataSheet_2.docx]
